# Diagnostic Accuracy of the Electrocardiography Criteria for Left Ventricular Hypertrophy (Cornell Voltage Criteria, Sokolow-Lyon Index, Romhilt-Estes, and Peguero-Lo Presti Criteria) Compared to Transthoracic Echocardiography

**DOI:** 10.7759/cureus.13883

**Published:** 2021-03-14

**Authors:** Nurseli Bayram, Haldun Akoğlu, Erkman Sanri, Sinan Karacabey, Melis Efeoğlu, Ozge Onur, Arzu Denizbasi

**Affiliations:** 1 Department of Emergency Medicine, Marmara University Pendik Education and Research Hospital, Istanbul, TUR; 2 Department of Emergency Medicine, Marmara University School of Medicine, Istanbul, TUR

**Keywords:** left ventricular hypertrophy, electrocardiography, echocardiography, sokolow-lyon index, cornell voltage index, romhilt-estes score system, peguero-lo presti criteria

## Abstract

Objective/Aim: We aimed to evaluate the diagnostic utility of the widely used left ventricular hypertrophy (LVH) electrocardiography (ECG) criteria (Cornell Voltage Criteria [CVC], Sokolow-Lyon Index [SLI], Romhilt-Estes [REC], and Peguero-Lo Presti [PLP] Criteria) compared with the left ventricular mass measured by echocardiography.

Methods: In this prospective diagnostic accuracy study, we screened all consecutive adults (18 to 65 years) who presented to our academic emergency department (ED) with increased blood pressure (≥130/85 mmHg) between January 2016 and January 2017, and we enrolled a convenience sample of 165 patients in our study. The attending emergency physician managed all patients as per their primary complaint. The consulting cardiologist performed a transthoracic echocardiogram (TTE) of the patient and calculated the left ventricular mass (LVM) according to the American Society of Echocardiography (ASE) formula. After completing the patient recruitment phase, researchers evaluated all ECGs and calculated scores for SLI, CVC, REC, and PLP. We used contingency tables to calculate the diagnostic utility metrics of all ECG criteria.

Results: The prevalence of LVH by TTE was 31.5%. CVC, SLI, REC, and PLP criteria correctly identified (true positive rate) abnormal LVM in only 3.9%, 1.9%, 9.6%, and 19.2% of the patients, respectively. CVC, SLI, REC score and PLP criteria performed poorly with extremely low sensitivities (3.9%, 1.9%, 10%, 19.2%) and poor accuracies (67.3%, 64.9%, 57.7%, 69.7%).

Conclusion: ECG voltage criteria's clinical utility in estimating LVM and LVH is low, and it should not be used for this purpose.

## Introduction

Left ventricular hypertrophy (LVH) is a compensatory mechanism, which is induced by the heart to compensate for the high arterial blood pressure (BP), and it is an early finding of hypertension (HTN). LVH develops in 15-20% of the HTN patients, and the presence of LVH is one of the best predictive factors for the cardiac outcome-independent from other risk factors of cardiovascular disorders (CVD) [1]. Studies showed that the presence of LVH in HTN patients increased the CVD risk five to 10 times [2-6]. Therefore, the early and definitive diagnosis of LVH is an essential factor regarding the decision on the HTN treatment [7]. 

Regarding the daily clinical practice, electrocardiography (ECG) is a routinely used, simple, cheap, and readily available method, appears in international guidelines, and the most commonly used screening tool for LVH [8]. The most frequently used ECG voltage criteria to estimate LVH are classical Sokolow-Lyon Index (SLI) and Cornell Voltage Criteria (CVC), and more recent Romhilt-Estes Criteria (REC) score and Peguero-Lo Presti Criteria [9-12]. However, despite their widespread acceptance and routine use in the clinical practice, the diagnostic accuracies of those criteria are low, especially with low sensitivity to rule-out and the presence of abnormally increased left ventricular mass (LVM) [12-14]. The classical paradigm of ECG diagnosis of LVH is based on the empirical finding of increased QRS voltage in cases of LVH, and a continuous effort is devoted to finding ECG criteria that agree best with LVH classification according to an ECG-independent standard such as transthoracic echocardiographic (TTE) LVH based on increased LVM. Recent guidelines suggested using LVM measured by 3D TTE (linear method) as an accurate method for estimating the degree of LVM anomaly that can precisely assess only by cardiac Magnetic Resonance Imaging (MRI) [15]. MRI is the gold standard in diagnosing increased LVM but expensive and impractical as a screening tool [15]. On the other hand, TTE can be used at the bedside, has a relatively short execution time, is a non-invasive method, and readily available. So, it became the most commonly preferred standard method in the diagnosis of LVH. However, the diagnostic utility of the classical and more recently suggested ECG voltage compared to TTE findings for detecting LVH is limited in the literature. Therefore, we aimed to compare the diagnostic accuracy of four ECG voltage criteria for detecting LVH using the LVM measured by 3D TTE as the reference standard.

## Materials and methods

Study design, population, and sample, inclusion and exclusion criteria

We conducted this prospective, observational, diagnostic accuracy study at an academic emergency department (ED) between January 2016 and 2017. All adult (age of 18 to 65 years) patients with a high BP reading at the triage (≥ 130/85 mmHg) of the ED were eligible for the study participation regardless of their primary complaint. A convenience sample was then derived from all consecutive patients who attended the ED during the shifts of the study researchers (n=171). Researchers evaluated the patients and reviewed their ECGs, and patients with the following criteria were planned to be excluded to form the final study sample (n=165): (1) left or right bundle branch block (n=6); (2) pacemaker (n=0); (3) medical history of an acute coronary syndrome (n=0); (4) unstable vital signs or coma status (n=0). This study was approved by the Institutional Ethics Board (Approval No: 09.2016.081), and written informed consent was obtained from each participant.

Data Collection

The attending emergency physician managed all patients as per their primary complaint. All study patients were referred to the consulting cardiologist of the day to perform a TTE, with a specific request to calculate LVM according to the American Society of Echocardiography (ASE) formula [15]. Demographic data were retrieved from the hospital information system and patient data files. ECGs were electronically recorded and physically filed for evaluation in the future. TTE recordings were also recorded. 

Measurements

BP Measurement

BP was measured according to the 2013 European Society of Cardiology/European Society of Hypertension (ESH/ESC) Guidelines for the Management of the Arterial Blood Pressure, on both arms, after the patients had rested for five minutes while sitting and applying a cuff chosen according to the weight of patients [8]. The measurements were performed with a digital blood pressure device (UA-1020 Premier Blood Pressure Monitor; A&D Company Ltd., Abingdon, UK). The measurement was performed three times in five-minute intervals. The second and third measurements were carried out on the arm, which gave the higher BP at the first measurement. 2013 ESH/ESC guideline classified 130/85 mmHg BP as high-normal. Patients with a blood pressure ≥130/85 mmHg in all three measurements were included in the study. The third measurement was recorded in the case report form.

ECG Voltage Criteria (Index Tests)

A 12-derivation ECG examination was performed during the resting period (Nihon Kohden Cardiofax®) and recorded in patient files. After the completion of the patient recruitment, all ECGs were evaluated by researchers (a PGY4 Emergency Medicine resident, a consultant, and faculty in emergency medicine), and SLI [10], CVC [11], REC [16], and PLP [9] scores were assessed, calculated, and recorded (Table 1, 2, 3, 4) [9-12,16,17].

**Table 1:** Sokolow-Lyon index

Sokolow-Lyon Index (if any is present) [10]
S wave in V1 + R wave in V5 or V6 ≥ 35 mm
R wave in aVR ≥ 11 mm

**Table 2:** Cornell voltage index

Cornell voltage Index [11]
S wave in V3 + R wave in aVL > 28 mm (male)
S wave in V3 + R wave in aVL > 20 mm (female)

**Table 3:** Peguero-Lo Presti Criteria

Peguero-Lo Presti Criteria [9]
Female: S_D_ + SV_4_ ≥ 2.3 mV
Male: S_D_ + SV_4_ ≥ 2.8 mV
If S_D_ is in lead V4, double the S wave amplitude to obtain the S_D_ + SV_4_

**Table 4:** Romhilt-Estes score system LVH: left ventricular hypertrophy

Romhilt-Estes Score System [16]	Score
Voltage Criteria (if any is present)	3
R or S wave in limb leads ≥ 20 mm or	
S wave in V1 or V2 ≥ 30 mm or	
R wave in V5 or V6 ≥ 30 mm	
ST-T Abnormalities	
ST-T vector opposite to QRS without digitalis	3
ST-T vector opposite to QRS with digitalis	1
Normal ST-T vector	0
P wave anomaly	
Negative terminal P wave in V1 ≥ 1 mm in depth or 0.40 s in duration	3
Others	
Left axis deviation (QRS -30° or more)	2
Delayed intrinsicoid deflection in V5 or V6 (> 0.05 s)	2
QRS duration ≥ 0.90 s	2
3 points or less: no LVH 4 points: probable LVH 5 or more points: positive for LVH	

TTE (Reference Standard) 

Recent guidelines suggested using LVM measured by 3D TTE (linear method) as an accurate method for estimating the degree of LVM anomaly [15]. Transthoracic echocardiographic (TTE) examination was performed with Philips EPIQ 7 (Philips Medical Systems, Bothell, WA, USA) with an adult probe (Philips X5-1 xMatrix 3.5 MHz). The measurements were performed according to the updated recommendations of the ASE and the European Association of Cardiovascular Imaging [15]. The septal thickness (IVS), left ventricular posterior wall thickness (LVPWT), and left ventricular internal diameter (LVID) were measured. The measurements were performed at end-diastole (d). LVM was calculated with the ASE formula (LVM = 0.8 x (1.04 x [(IVSd + LVIDd + LVPWTd)3 - LVID3] + 0.6)) and expressed in grams [15]. Degrees of abnormality of LVM recommended by the ASE guideline for the linear method of TTE was used for classifications (Table 5). TTE was performed by the consulting cardiologist, who was blinded to the study but not to the ECG, and all the above calculations and measurements were performed at the same visit.

**Table 5:** Degrees of abnormality of left ventricular mass (LVM) measured by the linear method according to American Society of Echocardiography (ASE) guideline

Linear Method	Women (grams)	Men (grams)
Reference range	67-162	88-224
Mildly abnormal	163-186	225-258
Moderately abnormal	187-210	259-292
Severely abnormal	≥211	≥293

Statistical analysis

Continuous variables were reported as medians and interquartile ranges (IQR) since they all had non-normal distributions. Categorical variables were declared with their frequencies and percentages. Chi-squared, Fisher's exact, and Mann Whitney U tests were used for group comparisons. Contingency tables were used to calculate the diagnostic utility metrics of the ECG criteria. An open-source statistical analysis package based on R was used for all analyses with a 2-sided significance threshold of 5% (Jamovi v1.0.7.0, The Jamovi project, 2019, www.jamovi.org). STARD 2015 guidelines for reporting diagnostic accuracy studies were used as a reference while preparing for this report [18].

## Results

A total of 165 patients were included in this study. The median age of the study sample was 50 years (IQR: 42 - 58), and 80 (48.5%) of the patients were female (Table 6). Since the classification of the degree of LVM abnormality is defined according to sex, we compared the demographics, high BP prevalence, and BP values between male and female patients (Table 6). Male patients were significantly younger (47 vs. 52 years), but the prevalence of high BP (57.6% vs. 50%) and baseline BP levels were similar. The prevalence of LVH by TTE was 31.5% in our study population. 

**Table 6:** Baseline variables according to sex P values were calculated using the Mann-Whitney U test. Bold values indicate statistical significance. IQR: interquartile range, SBP, DBP and MAP: Systolic, Diastolic and Mean blood pressure, LVM: Left ventricular mass, HTN: Hypertension.

	Total	Sex		
	(n=165)	Male (n=85)	Female (n=80)	P
Age (years), median (IQR)	50 (42-58)	47 (40-56)	52 (45-59)	0.045
SBP (mmHg), median (IQR)	162 (151-183)	163 (151-181)	162 (150-183)	0.775
DBP (mmHg), median (IQR)	97 (91-104)	97 (91-104)	96 (91-102)	0.647
MAP (mmHg), median (IQR)	119.7 (110.3-130.3)	120.3 (110.3-128)	118.3 (110.3-132.1)	0.782
LVM (gram), median (IQR)	169 (142-200)	189 (155-213)	156 (142-186)	<0.001
HTN, n (%)	89 (53.9)	49 (57.6)	40 (50.0)	0.325

The contingency tables of each ECG voltage criteria compared to the degree of LVM abnormality are presented in Table 7, and the diagnostic utility metrics are shown in Table 8. CVC, SLI, REC, and PLP criteria correctly identified (true positive rate) abnormal LVM in only 3.9%, 1.9%, 9.6%, and 19.2% of the patients, respectively. CVC, SLI, REC score and PLP criteria performed poorly with extremely low sensitivities (3.9%, 1.9%, 10%, 19.2%) and poor accuracies (67.3%, 64.9%, 57.7%, 69.7%). 

**Table 7:** Contingency tables of ECG criteria according to the degree of LVM abnormality as defined by American Society of Echocardiography (ASE) CVC: Cornell Voltage Criteria, SLI: Sokolow Lyon Index, REC: Romhilt-Estes Criteria Score, PLP: Peguero-Lo Presti Criteria, LVH: Left ventricular hypertrophy, LVM: Left ventricular mass. Percentages are for columns. Chi-squared tests, p values: CVC: 0.785; SLI: 0.447; REC 0.201; PLP 0.07

			LVM ASE Class, n (%)				
LVH according to ECG criteria			Normal LVM	Abormal LVM	Abnormal LVM		
n (%), (N=165)			(n=113)	(n=52)	Mild (n=27)	Moderate (n=13)	Severe (n=12)
CVC		6 (3.6)	4 (3.5)	2 (3.9)	1 (3.7)	1 (7.7)	0
SLI		8 (4.9)	7 (6.4)	1 (1.9)	0	1 (7.7)	0
REC	Probable	1 (0.1)	0	1 (1.9)	1 (3.7)	0	0
	LVH	29 (17.6)	24 (21.2)	5 (9.6)	2 (7.4)	1 (7.7)	2 (16.7)
PLP		18 (10.9)	8 (7.1)	10 (19.2)	6 (22.2)	2 (15.4)	2 (16.7)

**Table 8:** Comparison of the diagnostic utilities of 3 ECG voltage criteria for the detection of any abnormality in left ventricular mass (LVM) measured by echocardiography CVC: Cornell Voltage Criteria, SLI: Sokolow Lyon Index, REC: Romhilt-Estes Criteria Score, PLP: Peguero-Lo Presti Criteria. CI: Confidence Interval, AUC: Area Under the Curve, LR: Likelihood Ratio A score of 4 and above is accepted as LVH for Romhilt-Estes Criteria (REC) Score.

	AUC (%) (95% CI)	Sensitivity (%) (95% CI)	Specificity (%) (95% CI)	+LR (95% CI)	-LR (95% CI)
CVC	67.3 (59.6 – 74.4)	3.9 (0.5 – 13.2)	96.5 (91.2 – 99.0)	1.1 (0.2 – 5.7)	1 (0.9 – 1.1)
SLI	64.9 (57.0 – 72.1)	1.9 (0.05 – 10.3)	93.8 (87.7 – 97.5)	0.3 (0.04 – 2.5)	1.1 (1 – 1.1)
REC Score	57.7 (49.7 – 65.4)	9.6 (3.3 – 21.8)	78.8 (70.1 – 85.9)	0.5 (0.2 – 1.2)	1.1 (1 – 1.3)
PLP	69.7 (62.1 – 76.6)	19.2 (9.6 – 32.5)	92.9 (86.5 – 96.9)	2.7 (1.1 – 6.5)	0.9 (0.8 - 1)

## Discussion

In this study, we assessed the clinical utility of four established ECG voltage criteria to estimate LVH in patients presented to the triage of an ED with elevated BP. We found that ECG voltage criteria are not suitable for estimating the increased LVM. 

According to previous studies, the sensitivities of CVC and SLI were low, and specificities were high [7,19,20]. Recently, Ricciardi et al. retrospectively reviewed and compared LVH ECG voltage criteria with TTE in 2134 hospitalized patients and reported the area under the curve (AUC) of CVC as 67.8% with a sensitivity of 31% and specificity of 88%, and AUC of SLI as 61.4 with a sensitivity of 24.8% and specificity of 91.6% [19]. Similarly, Su et al. reviewed the ECG of 539 male military members for LVH ECG voltage criteria compared to TTE. They reported the AUCs of CVC and SLI as 0.66 and 0.54 [20]. In a systematic review by Vanezis et al., the weighted mean sensitivities of CVC and SLI in African-origin/White population were 31.2%/26.5% and 32.9%/18.2%, weighted mean specificities were 86.2%/87.4% and 72.1%/88.9%, respectively [1]. Elffers et al. reported AUC, sensitivity, and specificity of SLI and CVC as 58%, 16%, and 90%, and 65%, 28%, and 90%, respectively, in their population-based cohort study of 6671 individuals entitled The Netherlands Epidemiology of Obesity study (NEO) [21]. In a retrospective cohort of 21,286 patients, Kwon et al. studied an artificial intelligence algorithm for detecting LVH and reported the sensitivities of CVC (by computer interpretation), SLI, and REC (by cardiologist interpretation) as 34.5%, 34.5%, and 28.4%; and specificities as 93.6%, 89.6%, and 95.1%, respectively [22]. Our findings were similar to those studies summarized above with lower sensitivities. 

Recently, Peguero and Lo Presti proposed a new set of criteria and claimed that this criterion has higher sensitivity and specificity than other voltage criteria in estimating LVM [9]. Shao et al. validated this criterion in a Chinese cohort of 235 hospitalized HTN patients [23]. In their cohort, 116/235 (49.3%) of the patients had confirmed LVH. They reported an AUC of 77.2% for males and 83.2% for females, which is higher than the AUC we found in our cohort. Since their LVH prevalence was high, their reported sensitivities were also higher as expected. 

Ricciardi et al. argued the logic in expecting a 2D evaluation like ECG to be in complete agreement with a 3D problem like LVH, and proposed a change in the paradigm of ECG analysis, which we also agree [19]. We think that ECG data in its current form is inefficient and incomplete for estimating LVH, and a paradigm shift is needed especially at the bedside while evaluating the ECG of an undifferentiated patient in the ED. LVH or LVM cannot be estimated successfully by using any voltage criteria, and ED physicians should not rely on ECG to rule-out or rule-in this diagnosis. 

Even though this study's sample size is smaller compared with the studies mentioned above, the inclusion of REC and PLP for comparison, use of 3D TTE as a reference, and studying the validity in acute ED patients were the substantial aspects of this study.

Limitations

First, in this study, the LVM of the patients measured by TTE was not controlled for body mass index and body surface area of the patients. Second, the study population includes patients with high-normal BP readings in the triage area of the ED. Therefore, findings could not be extrapolated to all ED patients or patients with hypertension. Third, the high LVH prevalence rate of 31.5% could have increased the sensitivities we calculated. However, we found all sensitivities to be lower despite the high prevalence of LVH, which confirms our findings. Fourth, we used TTE as the reference standard, not cardiac MRI, since it is the most commonly used clinical practice method and is known to have good agreement with MRI findings.

## Conclusions

One of the causes of LVH is HTN. LVH may be a compensatory mechanism, which is induced by the heart to compensate for the BP, and it is an early finding of HTN. In this study, we assessed the clinical utility of four established ECG voltage criteria to estimate LVH in patients presented to the triage of an ED with elevated BP. We found that ECG voltage criteria may not be suitable for estimating the increased LVM. ECG voltage criteria' clinical utility in estimating LVM and LVH is low, and it should not be used for this purpose.
